# Supplementary material for: Giardiasis Alters Intestinal Fatty Acid Binding Protein (I-FABP) and Plasma Cytokines Levels in Children in Brazil
Source: Pathogens. 2019 Dec 19;9(1):7. doi: 10.3390/pathogens9010007 (PMC7169386; doi:10.3390/pathogens9010007)
Supplement: Supplementary file 1 [file pathogens-09-00007-s001.pdf]

Supplementary Table S1 – Cytokines and chemokines profile in plasma of children infected by *Giardia lamblia*

Cytokines	Preschoolers				
Chemokines	*Giardia* Positive (MFI)		*Giardia* Negative (MFI)		p
IFN-γ	10	[7.2 – 14]	10.0	[5.2 – 22.5]	ns
TNF	40	[30 – 50]	33.5	[27.5 – 54.5]	ns
IL-1β	2.0	[0.0 – 6.5]	4.5	[1.3 – 10.7]	ns
IL-2	0.0	[0.0 – 18.5]	0.0	[0.0 – 0.0]	ns
IL-4	0.0	[0.0 – 0.0]	0.0	[0.0 – 0.0]	ns
IL-5	0.0	[0.0 – 11]	7.0	[3.6 – 11.7]	0.09
IL-6	11	[4.7 – 17]	12.7	[6.5 – 16.2]	ns
IL-7	8.0	[4.7 – 10.7]	6.0	[4.2 – 7.5]	ns
IL-8	98	[76.2 – 133]	241	[191 – 459]	0.003
IL-10	16	[8.0 – 25.5]	10.5	[9.0 – 13.5]	*0.08
IL-12p40	0.0	[0.0 – 6.0]	2.5	[0.2 – 4.0]	ns
IL-13	5.5	[0.0 – 7.2]	4.5	[3.2 – 5.7]	ns
IL-17	12	[5.5 – 23.5]	8.5	[5.3 – 11.5]	*0.05
MCP-1	517	[309 – 562]	336	[116 – 1,234]	ns
MIP-1β	297	[218 – 732]	373	[240 – 835]	ns

IFN: interferon, IL: interleukin, TNF: tumor necrosis factor, MCP-1: monocyte chemoattractant protein-1, MIP-1β: macrophage inflammatory protein-1β. Data were expressed as Median Fluorescence Intensity (MFI). The results are presented as median [interquartile range 25-75%]. Statistical analysis was performed using no parametric Mann-Whitney test or *Student t –test.
